# Supplementary material for: Glia phagocytose neuronal sphingolipids to infiltrate developing synapses
Source: bioRxiv. 2025 Apr 22:2025.04.14.648777. Preprint. [Version 2] doi: 10.1101/2025.04.14.648777 (PMC12045345; doi:10.1101/2025.04.14.648777)
Supplement: 1 [file NIHPP2025.04.14.648777v2-supplement-1.pdf]

bioRxiv preprint doi: https://doi.org/10.1101/2025.04.14.648777; this version posted April 22, 2025. The copyright holder for this preprint (which was not certified by peer review) is the author/funder, who has granted bioRxiv a license to display the preprint in perpetuity. It is made available under aCC-BY 4.0 International license.

.

Supplemental Figures and Figure Legends

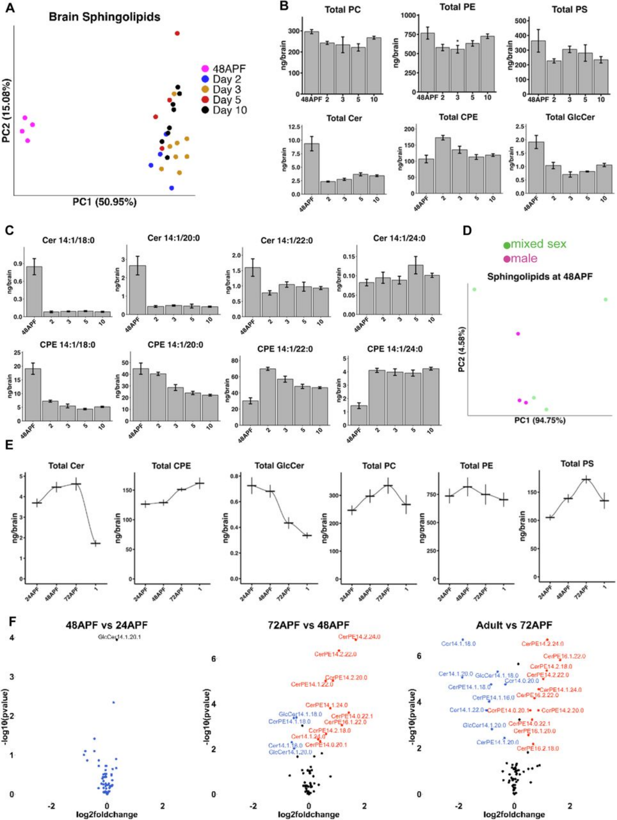

A
Brain Sphingolipids
48APF
Day 2
Day 3
Day 5
Day 10
PC2 (15.08%)
PC1 (50.95%)
B
Total PC
Total PE
Total PS
Total Cer
Total CPE
Total GlcCer
ng/brain
48APF
C
Cer 14:1/18:0
Cer 14:1/20:0
Cer 14:1/22:0
Cer 14:1/24:0
CPE 14:1/18:0
CPE 14:1/20:0
CPE 14:1/22:0
CPE 14:1/24:0
D
mixed sex
male
Sphingolipids at 48APF
PC2 (4.58%)
PC1 (94.75%)
E
Total Cer
Total CPE
Total GlcCer
Total PC
Total PE
Total PS
24APF
48APF
72APF
F
48APF vs 24APF
72APF vs 48APF
Adult vs 72APF
-log10(pvalue)
log2foldchange

bioRxiv preprint doi: https://doi.org/10.1101/2025.04.14.648777; this version posted April 22, 2025. The copyright holder for this preprint (which was not certified by peer review) is the author/funder, who has granted bioRxiv a license to display the preprint in perpetuity. It is made available under aCC-BY 4.0 International license.

Figure S1, related to Figure 1.

(A) PCA analysis of sphingolipids from control brains dissected during development and 48h APF (magenta) and adult ages.

(B) Total phospholipids and sphingolipids measured at 48h APF and multiple adult ages, quantified in ng/brain.

(C) Developmentally modulated sphingolipid levels in ng/brain at 48h APF and adult ages.

(D) PCA analysis at 48h APF of male (magenta) and mixed sex (green) brains.

(E) Developing brain total sphingolipids and phospholipids (ng/brain).

(F) Volcano plots between the 4 developmental timepoints (48h APF versus 24h APF; 72h APF versus 48h APF; and day 1 versus 72h APF).

Lipidomics in E-F represent 8 tubes of 15 brains per each timepoint, lipidomics in A-D are 8 tubes of 15 brains per timepoint for day 3 and day 10, and 4 tubes of 15 brains per timepoint for all other ages. Data are represented as mean ± SEM.
* $p < 0.05$, ** $p < 0.01$, *** $p < 0.001$, **** $p < 0.0001$

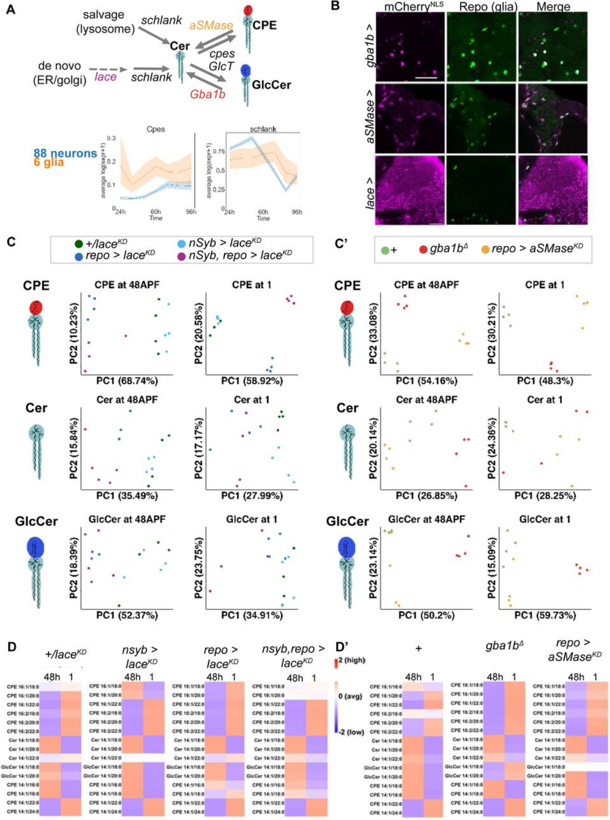
A
salvage (lysosome)
schlank
aSMase
CPE
Cer
cpes
GlcT
de novo (ER/golgi)
lace
schlank
Gba1b
GlcCer
88 neurons
6 glia
Cpes
schlank
average log(expr+1)
Time
24h
60h
96h
B
mCherry^NLS
Repo (glia)
Merge
gba1b >
aSMase >
lace >
C
+/lace^KD
repo > lace^KD
nSyb > lace^KD
nSyb, repo > lace^KD
C'
+
gba1b^Δ
repo > aSMase^KD
CPE
CPE at 48APF
CPE at 1
PC1 (68.74%)
PC2 (10.23%)
PC1 (58.92%)
PC2 (20.58%)
PC1 (54.16%)
PC2 (33.08%)
PC1 (48.3%)
PC2 (30.21%)
Cer
Cer at 48APF
Cer at 1
PC1 (35.49%)
PC2 (15.84%)
PC1 (27.99%)
PC2 (17.17%)
PC1 (26.85%)
PC2 (20.14%)
PC1 (28.25%)
PC2 (24.36%)
GlcCer
GlcCer at 48APF
GlcCer at 1
PC1 (52.37%)
PC2 (18.39%)
PC1 (34.91%)
PC2 (23.75%)
PC1 (50.2%)
PC2 (23.14%)
PC1 (59.73%)
PC2 (15.09%)
D
+/lace^KD
nsyb > lace^KD
repo > lace^KD
nsyb,repo > lace^KD
48h
1
D'
+
gba1b^Δ
repo > aSMase^KD
2 (high)
0 (avg)
-2 (low)

bioRxiv preprint doi: https://doi.org/10.1101/2025.04.14.648777; this version posted April 22, 2025. The copyright holder for this preprint (which was not certified by peer review) is the author/funder, who has granted bioRxiv a license to display the preprint in perpetuity. It is made available under aCC-BY 4.0 International license.

Figure S2, related to Figure 2.

(A) Simplified diagram of enzymes in sphingolipid biosynthetic and salvage (catabolic) pathways. scRNAseq data replotted from[74], 88 neural cluster average is shown in blue, and glial cluster average depicted in orange, from 24h to 96h APF. Data are mean ± SEM.

(B) *CRIMIC-GAL4* driven *nls-mCherry* using *Gba1b-GAL4*, *aSMase-GAL4,* or *lace-GAL4* (magenta), co-stained with the glial marker repo (green). *Gba1b*-expressing cells were 97% repo+, *aSMase*-expressing cells were 91% repo+, and *lace*-expressing cells were 8% repo+ (n = 5 brains each genotype). Scale bar = 20µm.

(C) PCA analysis of CPE, Cer, and GlcCer at 48h APF and day 1 for biosynthetic manipulations targeting *lace* by glial (*repo-GAL4*, blue), neural (*nSyb-GAL4*, light red), or combined neural and glial drivers (*nSyb-GAL4, repo-GAL4,* light purple) versus controls (*+/lace-RNAi*, light green). (C'). PCA analysis of CPE, Cer, and GlcCer at 48h APF and day 1 for catabolic manipulations (*gba1b*$^{\Delta}$, red) and *aSMase* knockdown in glia (*repo-GAL4* > SMase^{KD}, orange), versus controls (green).

(D-D') Z-scores of major developmentally regulated sphingolipids across time in biosynthetic (D) and catabolic (D') manipulations reveals that the global patterns of developmental changes in sphingolipids is relatively robust across genotypes.

n= 8 tubes of 15 brains per each timepoint for C-D.
* p < 0.05, ** p < 0.01, *** p < 0.001, **** p < 0.0001

bioRxiv preprint doi: https://doi.org/10.1101/2025.04.14.648777; this version posted April 22, 2025. The copyright holder for this preprint (which was not certified by peer review) is the author/funder, who has granted bioRxiv a license to display the preprint in perpetuity. It is made available under aCC-BY 4.0 International license.

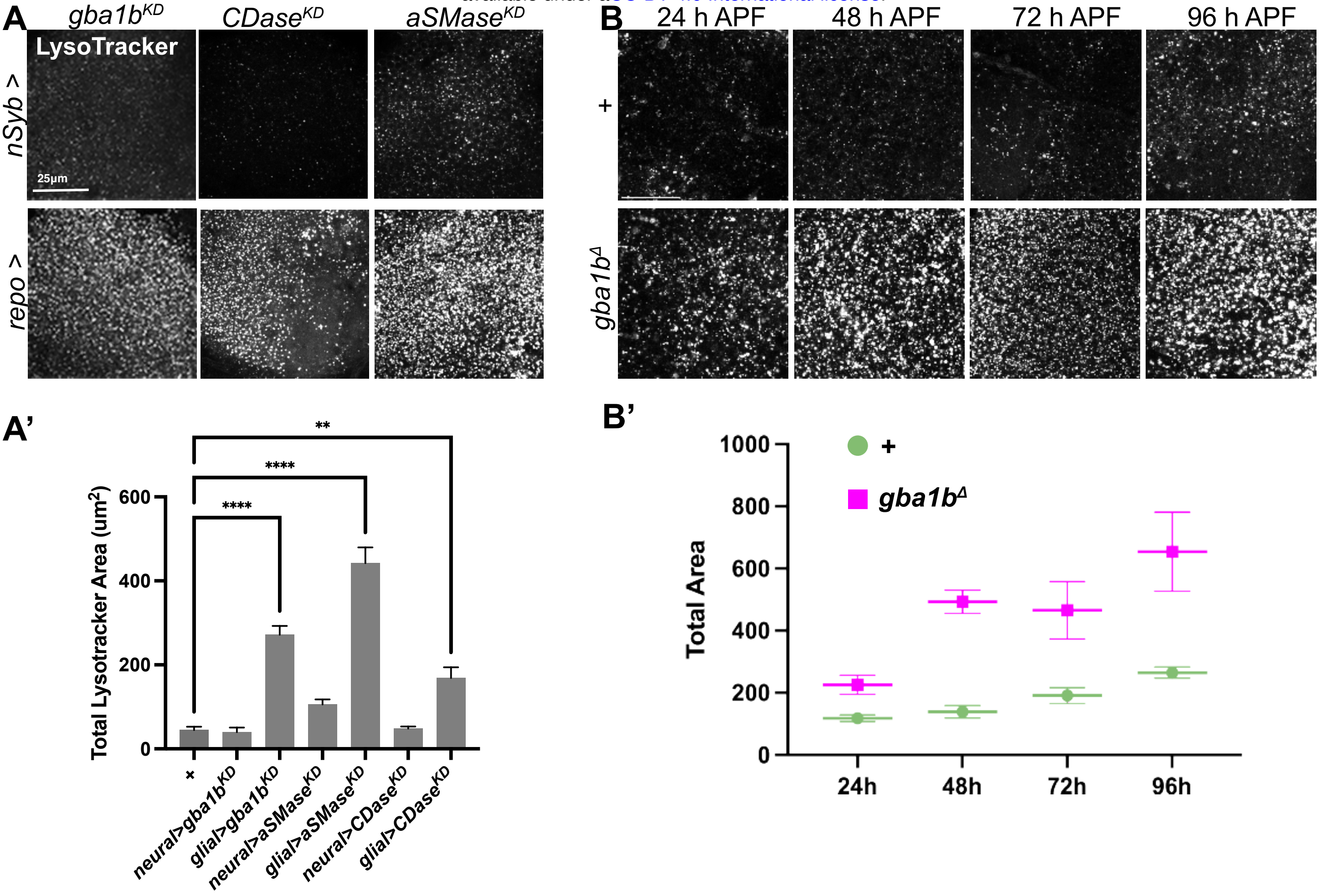

bioRxiv preprint doi: https://doi.org/10.1101/2025.04.14.648777; this version posted April 22, 2025. The copyright holder for this preprint (which was not certified by peer review) is the author/funder, who has granted bioRxiv a license to display the preprint in perpetuity. It is made available under aCC-BY 4.0 International license.

Figure S3, related to Figure 3.

(A-A') Confocal images of lysotracker staining (white), with maximum intensity projections of optic lobes from neural (*nSyb-GAL4*) or glial (*repo-GAL4*) knockdowns of sphingolipid catabolic enzymes. A', quantification of lysotracker area. Scale bar = 25µm.

(B') Timecourse of lysotracker from central brains in *gba1b*$^{\Delta}$ and control brains across pupal development, quantified in B' (green = control, magenta = *gba1b*$^{\Delta}$). Scale bar = 20µm.

Data are represented as mean ± SEM. n > 10 brains all experiments.
* p < 0.05, ** p < 0.01, *** p < 0.001, **** p < 0.0001

bioRxiv preprint doi: https://doi.org/10.1101/2025.04.14.648777; this version posted April 22, 2025. The copyright holder for this preprint (which was not certified by peer review) is the author/funder, who has granted bioRxiv a license to display the preprint in perpetuity. It is made available under aCC-BY 4.0 International license.

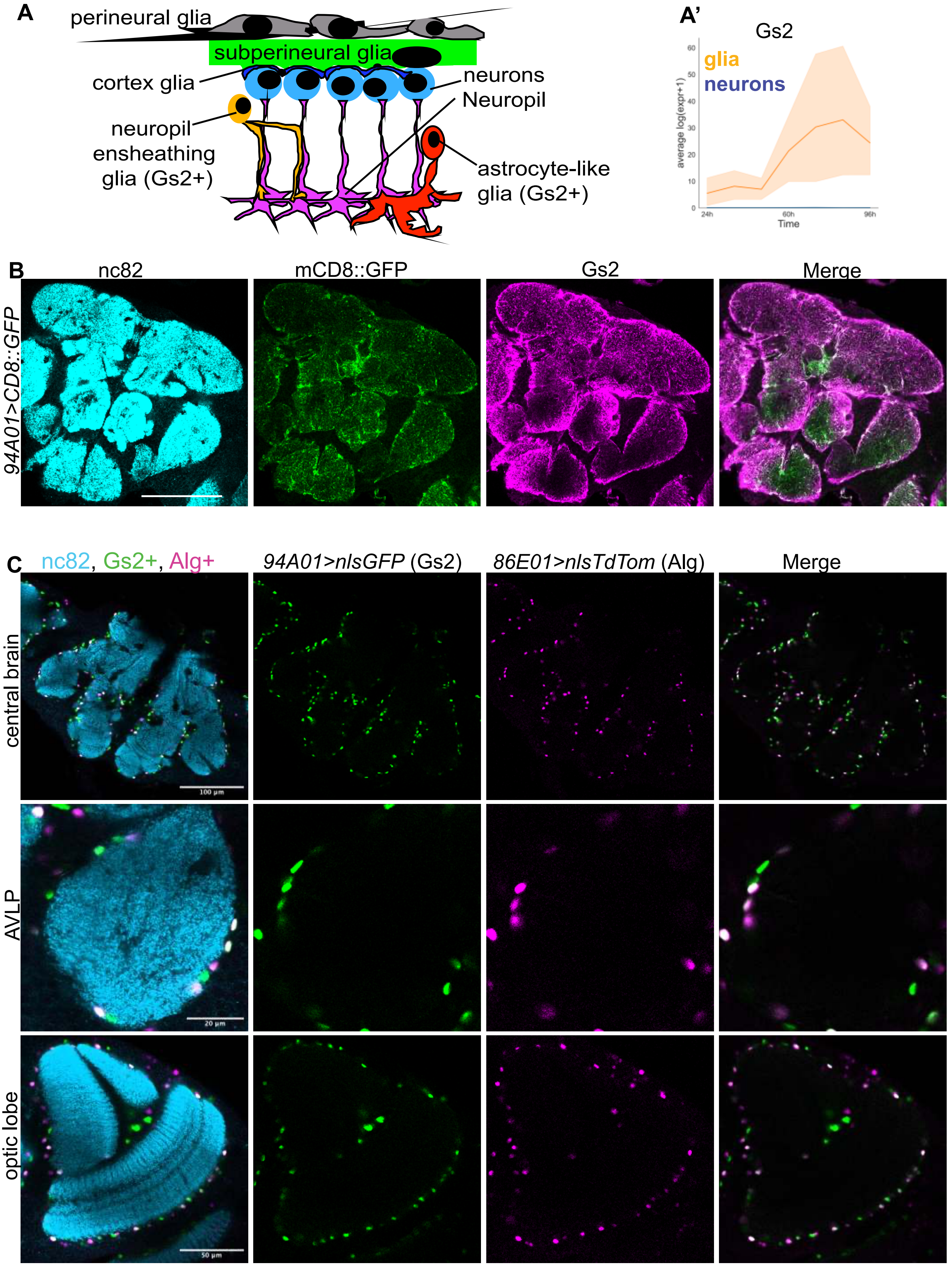

bioRxiv preprint doi: https://doi.org/10.1101/2025.04.14.648777; this version posted April 22, 2025. The copyright holder for this preprint (which was not certified by peer review) is the author/funder, who has granted bioRxiv a license to display the preprint in perpetuity. It is made available under aCC-BY 4.0 International license.

Figure S4, related to Figure 4.

(A) Schematic of fly brain neurons and glia, with Gs2+ glia encompassing neuropil types composed of ensheathing (orange) and astrocyte-like (red) glia[85].
(A)' Gs2 expression in scRNAseq datasets from[74], with glia (orange) exclusively expressing Gs2, with expression increasing from 48h APF.

(B) Gs2 enhancer trap (*GMR94A01-GAL4*) expressing *UAS-CD8::GFP* (green), stained with the neuropil marker nc82 (light blue) and Gs2 (magenta) in day 1 brains. Gs2+ GFP-labeled membranes are decorated by Gs2; note the absence of cortex and barrier signal. Scale bar = 100μm. n = 5 brains.

(C) Day 1 adult brains labelingr Gs2+ cells with *GMR94A01-GAL4 >UAS-nls-STINGER* (green) and astrocyte-like glia (ALG) labeled with *GMR86E01-LexA > LexAop-nls-TdTom* (magenta) in central brain (scale bar = 100μm), AVLP (scale bar = 20μm), and optic lobe (scale bar = 50μm). Neuropil is stained with nc82 (light blue). n = 3 brains.

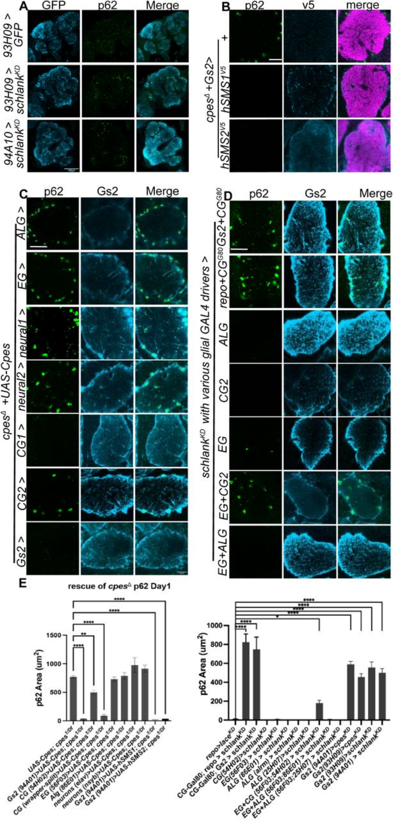

A
GFP
p62
Merge
93H09 > GFP
93H09 > schlank^KD
94A10 > schlank^KD
B
p62
v5
merge
cpes^Δ +Gs2>
+
hSMS1^V5
hSMS2^V5
C
p62
Gs2
Merge
cpes^Δ +UAS-Cpes
ALG >
EG >
neural1 >
neural2 >
CG1 >
CG2 >
Gs2 >
D
p62
Gs2
Merge
schlank^KD with various glial GAL4 drivers >
Gs2+CG^G80
repo+CG^G80
ALG
CG2
EG
EG+CG2
EG+ALG
E
rescue of cpes^Δ p62 Day1
p62 Area (um^2)
UAS-Cpes; cpes^1/Df
Gs2 (94A01)>UAS-Cpes; cpes^1/Df
CG (54H02)>UAS-Cpes; cpes^1/Df
CG (wrapper-split)>UAS-Cpes; cpes^1/Df
EG (56F03)>UAS-Cpes; cpes^1/Df
Alg (86E01)>UAS-Cpes; cpes^1/Df
neurons (elav)>UAS-Cpes; cpes^1/Df
neurons (nsyb)>UAS-Cpes; cpes^1/Df
Gs2 (94A01)>UAS-hSMS1; cpes^1/Df
Gs2 (94A01)>UAS-hSMS2; cpes^1/Df
p62 Area (um^2)
repo>lace^KD
CG-Gal80; repo > schlank^KD
CG-Gal80; Gs2 > schlank^KD
CG(54H02)>schlank^KD
EG(56F03) > schlank^KD
ALG (86E01) > schlank^KD
ALG (alrm) > schlank^KD
ALG (25H07)>schlank^KD
EG+CG (56F03;54H02) > schlank^KD
EG+ALG (56F03;86E01) > schlank^KD
EG+ALG (56F03;25H07) > schlank^KD
Gs2 (94A01)>cpes^KD
Gs2(93H09)>cpes^KD
Gs2 (93H09)>schlank^KD
Gs2 (94A01) > schlank^KD

bioRxiv preprint doi: https://doi.org/10.1101/2025.04.14.648777; this version posted April 22, 2025. The copyright holder for this preprint (which was not certified by peer review) is the author/funder, who has granted bioRxiv a license to display the preprint in perpetuity. It is made available under aCC-BY 4.0 International license.

Figure S5, related to Figure 5.

(A) Removing *schlank* (Ceramide Synthase) in Gs2 glia with two independent drivers (*GMR94A01-GAL4* or *GMR93H09-GAL4*) caused autonomous p62 accumulation (green) in GFP-labeled Gs2 membranes (light blue). Scale bar = 100 μm.

(B) Expressing *hSMS1::v5* or *hSMS2::v5* in Gs2+ glia with *GMR94A01-GAL4* in *cpes* nulls rescued p62 (green). V5 (blue) was expressed in a pattern consistent with the predicted subcellular compartment of these enzymes (golgi for SMS1, which appears punctate; plasma membrane for SMS2). Magenta shows nc82 neuropil in the AVLP. Scale bar = 20 μm.

(C) Glial or neuronal *GAL4* drivers re-expressing *UAS-Cpes* in attempted rescue of the *cpes* null mutant accumulation of p62 (green) in Gs2+ glia (light blue) in the AVLP. ALG = astrocyte-like glia (*GMR86E01-GAL4*); EG = ensheathing glia *(GMR56F03-GAL4*); neural1 = *elav-GAL4*; neural2 = *nSyb-GAL4*; CG1 = cortex glia (*cortex-split-GAL4*); CG2 = cortex glia (*GMR54H02-GAL4*); Gs2 = *94A01-GAL4*. Notably, Gs2 drivers fully rescued the p62 phenotype, while cortex glia drivers either fully rescued (*ctx-split*) or partially rescued (*GMR54H02*) p62 levels. Compensatory interactions between cortex glia and neuropil glia have been observed recently[154]. Scale bar = 20 μm.

(D) Staining for p62 (green) in Gs2+ glia (light blue) by glial driver combinations crossed to *schlank-RNAi*, including *Gs2-GAL4* or *repo-GAL4* with GAL80 produced in cortex glia (ctx^{G80}) to exclude leaky GAL4 expression in cortex glia. Outside of *Gs2-GAL4* or *repo-GAL4*, only the combined ensheathing glia + cortex glia knockdown (EG+CG > $schlank^{KD}$ using *GMR56F03-GAL4; GMR54H02-GAL4*) caused a partial p62 phenotype. Scale bar = 20 μm.

(E) Quantification of p62 aggregates in AVLP from genotypes in A-D. Data are represented as mean ± SEM. n > 10 brains per condition. * $p < 0.05$, ** $p < 0.01$, *** $p < 0.001$, **** $p < 0.0001$

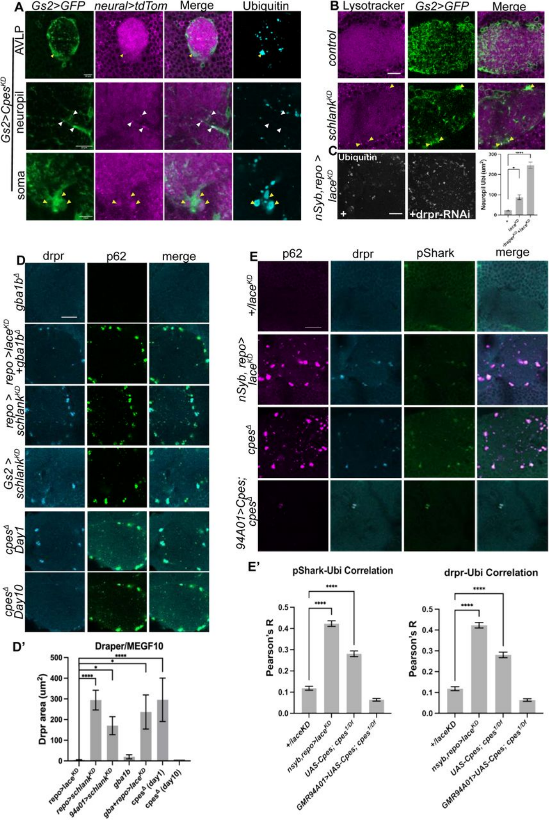
A
Gs2>GFP
neural>tdTom
Merge
Ubiquitin
Gs2>Cpes^KD
AVLP
neuropil
soma
B
Lysotracker
Gs2>GFP
Merge
control
schlank^KD
C
nSyb, repo > lace^KD
Ubiquitin
+
+drpr-RNAi
Neuropil Ubi (um²)
lace^KD
draper^KD+lace^KD
D
drpr
p62
merge
gba1b^Δ
repo >lace^KD +gba1b^Δ
repo > schlank^KD
Gs2 > schlank^KD
cpes^Δ Day1
cpes^Δ Day10
D'
Draper/MEGF10
Drpr area (um²)
repo>lace^KD
repo>schlank^KD
94a01>schlank^KD
gba1b
gba+repo>lace^KD
cpes^Δ (day1)
cpes^Δ (day10)
E
p62
drpr
pShark
merge
+/lace^KD
nSyb, repo> lace^KD
cpes^Δ
94A01>Cpes; cpes^Δ
E'
pShark-Ubi Correlation
drpr-Ubi Correlation
Pearson's R
+/laceKD
nsyb,repo>lace^KD
UAS-Cpes; cpes^1/Df
GMR94A01>UAS-Cpes; cpes^1/Df

bioRxiv preprint doi: https://doi.org/10.1101/2025.04.14.648777; this version posted April 22, 2025. The copyright holder for this preprint (which was not certified by peer review) is the author/funder, who has granted bioRxiv a license to display the preprint in perpetuity. It is made available under aCC-BY 4.0 International license.

Figure S6, related to Figure 6.

(A) Gs2 (green) and neuronal membranes (magenta) in the AVLP stained with ubiquitin (blue) in Gs2-glial knockdowns of *cpes* (scale bar = 20 µm), with zooms into neuropil (scale bar = 10 µm), and soma (scale bar = 5 µm). White arrows mark ubiquitin-, neuronal+ inclusions in glial membranes. Yellow arrows indicate ubiquitin+, weakly neuronal+ inclusions.

(B) Lysotracker staining of Gs2 glial membranes in AVLP in controls and *schlank* knockdowns, with smaller lysotracker signals evident in Gs2 soma (arrows). Scale bar = 20 µm.

(C) Removing *draper* worsens ubiquitin accumulation in brains with *lace* removed in both neurons and glia using *nSyb-GAL4, repo-GAL4*. Scale bar = 20 µm.

(D-D') Draper (light blue) accumulates in day 1 AVLP brains from genotypes that blockade CPE biosynthesis. Note that draper is lost from these structures by day 10 in *cpes* nulls. D', quantification of draper accumulation. Scale bar = 20 µm.

(E-E') pShark staining in AVLP correlates with draper and p62 in genotypes with a blockade in CPE biosynthesis. E', quantification. Scale bar = 20 µm.

Data are represented as mean ± SEM. n > 7 brains per condition.
* $p < 0.05$, ** $p < 0.01$, *** $p < 0.001$, **** $p < 0.0001$

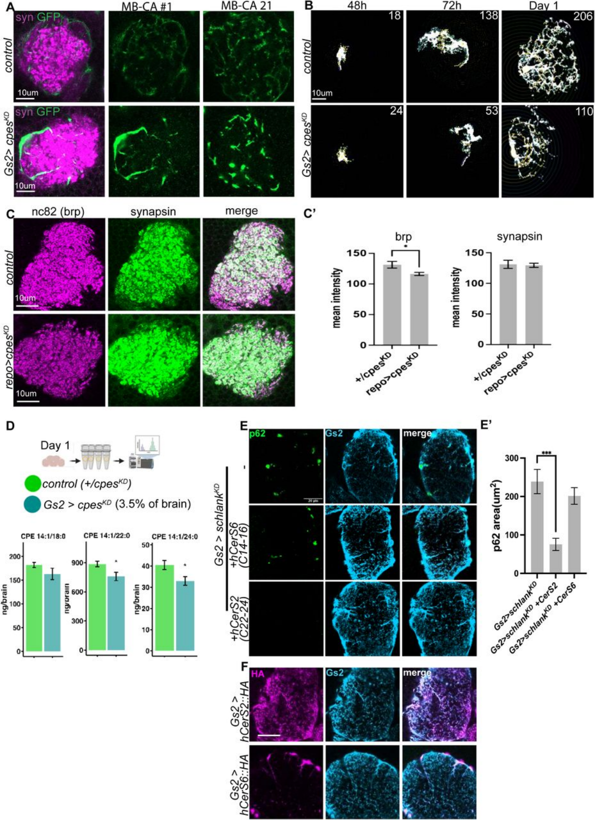
A
syn GFP
MB-CA #1
MB-CA 21
control
10um
Gs2> cpes^KD
B
48h
72h
Day 1
18
138
206
24
53
110
C
nc82 (brp)
synapsin
merge
repo>cpes^KD
C'
brp
synapsin
mean intensity
+/cpes^KD
repo>cpes^KD
D
Day 1
control (+/cpes^KD)
Gs2 > cpes^KD (3.5% of brain)
CPE 14:1/18:0
CPE 14:1/22:0
CPE 14:1/24:0
ng/brain
E
p62
Gs2
merge
Gs2 > schlank^KD
+hCerS6 (C14-16)
+hCerS2 (C22-24)
E'
p62 area(um²)
Gs2>schlank^KD
Gs2>schlank^KD +CerS2
Gs2>schlank^KD +CerS6
F
HA
Gs2 > hCerS2::HA
Gs2 > hCerS6::HA

bioRxiv preprint doi: https://doi.org/10.1101/2025.04.14.648777; this version posted April 22, 2025. The copyright holder for this preprint (which was not certified by peer review) is the author/funder, who has granted bioRxiv a license to display the preprint in perpetuity. It is made available under aCC-BY 4.0 International license.

Figure S7, related to Figure 7.

(A) Non-sparsely labeled Gs2 glial membranes (green) in control and *cpes-RNAi* mushroom body calyx (MB-CA). Syn = synapsin, neuropil marker in magenta. Aberrant thicker branches are observed in *cpes* knockdowns with *Gs2-GAL4*. Scale bars = 10 μm.

(B) Example Sholl analysis of sparsely labeled glial clones from Figure 7. Scale bar = 10 μm. Numbers in top right indicate number of intersections, a proxy for branching.

(C-C') Mushroom body calyx stained for brp (nc82, magenta) or synapsin (green) from controls or glia depleted of *cpes*. Brains were pooled and stained in the same primary and secondary well, then decoded for the presence of *cpes-RNAi* by p62 aggregates. Brp/nc82 levels were reduced, unlike synapsin. Scale bar = 10 μm.

(D) Lipidomics of controls ($+/cpes^{KD}$, green) versus *cpes* knockdown in Gs2+ glia (blue) from day 1 brains. CPE 14:1/22:0 and CPE 14:1/24:0 are decreased, despite the genetic manipulation only targetting only ~3.5% of the brain.

(E) Rescue of p62 (green) in *schlank/CerS* knockdown AVLPs in Gs2 glia (light blue) by heterologous expression of human hCerS2 or hCerS6. CerS2 generates C22-C24 VLCFA sphingolipids, while CerS6 generates C14-C16 sphingolipids. Scale bar = 20 μm.
(E') Quantification of p62 area.

(F) Localization of hCerS transgenes (magneta) by anti-HA staining when expressed in Gs2 glia (light blue). Scale bar = 20 μm.

Data are represented as mean ± SEM. n > 10 brains per genotype for A-C and E-F, and 4 tubes of 15 brains each for D.

* $p < 0.05$, ** $p < 0.01$, *** $p < 0.001$, **** $p < 0.0001$
